# The Association between Human Epididymis Secretory Protein 4 and Metabolic Syndrome

**DOI:** 10.3390/jcm11092362

**Published:** 2022-04-22

**Authors:** Peng-Fei Li, Yu-Jen Lin, Yao-Jen Liang, Wei-Liang Chen

**Affiliations:** 1 Division of Endocrinology and Metabolism, Department of Internal Medicine, Tri-Service General Hospital, National Defense Medical School, Taipei 114, Taiwan, China; milkfly198383@gmail.com; 2 Graduate Institute of Applied Science and Engineering, Fu-Jen Catholic University, New Taipei 242, Taiwan, China; 3 Division of Family Medicine, Department of Family and Community Medicine, Tri-Service General Hospital, Taipei 114, Taiwan, China; ronyowei@yahoo.com.tw; 4 School of Medicine, National Defense Medical Center, Taipei 114, Taiwan, China; 5 Graduate Institute of Applied Science and Engineering and Department & Institute of Life Science, Fu-Jen Catholic University, New Taipei 242, Taiwan, China; yjl@mail.fju.edu.tw; 6 Division of Geriatric Medicine, Department of Family and Community Medicine, Tri-Service General Hospital, Taipei 114, Taiwan, China; 7 Department of Biochemistry, National Defense Medical Center, Taipei 114, Taiwan, China

**Keywords:** human epididymis protein 4, 2 metabolic syndrome components, 3 carcinogenesis

## Abstract

Individuals with metabolic syndrome (MetS) are known to have an increased risk of carcinogenesis. Human epididymis protein 4 (HE4) is a tumor marker and prognostic factor for epithelial ovarian carcinoma (EOC) patients. However, no studies have evaluated the association between MetS and HE4 levels. This study aimed to evaluate the relationship between HE4 levels and MetS in the National Health and Nutrition Examination Survey (NHANES 2001–2002). This cross-sectional analysis assessed all five components of MetS and HE4 levels in 2104 females (age ≥20 years) from the NHANES dataset. MetS was defined according to the National Cholesterol Education Program Adult Treatment Panel III (NCEP ATPIII) criteria. The analysis indicated MetS in 593 individuals, and the β coefficient of their HE4 levels was 0.097 (95% CIs, 0.028–0.166, *p* = 0.006). Specifically, the β coefficients of the HE4 levels of participants with 1, 2, 3, and ≥4 features of MetS were 0.072 (95% confidence interval (CI): −0.015–0.159), 0.125 (95% CI: 0.030–0.220), 0.161 (95% CI: 0.053–0.270), and 0.242 (95% CI: 0.117–0.368), respectively, and all *p* values were <0.001. The *p*-value for the trend was <0.001. There was a significant association between the presence of MetS and HE4 levels. There were positive relationships between HE4 levels and an increased number of MetS components (with 1, 2, 3, and ≥4 features of MetS, all *p* values <0.001). Among the MetS components, low high-density lipoprotein levels and high triglyceride levels were independently associated with HE4 levels.

## 1. Introduction

Metabolic syndrome (MetS) is a clustering of hyperglycemia, hyperlipidemia, hypertension, and central obesity, as suggested by the National Cholesterol Education Program (NCEP). Many studies have associated MetS with cardiovascular diseases and type 2 diabetes mellitus (T2DM) [1,2]. Based on previous studies, chronic inflammation and insulin resistance were core mechanisms underlying the pathophysiology of MetS [3,4]. The prevalence of MetS in the US from 2011 to 2016 (aged ≥20 years) was 34.7% based on the National Health and Nutrition Examination Survey (NHANES) [5]. Owing to the rising that impaired energy metabolism and genomic instability might be the primary cause of cancer, many researchers have examined the association between MetS and cancer [6,7,8]. Numerous studies showed that oxidative stress, chronic inflammation, and angiogenesis in MetS individuals led to carcinogenesis [9,10]. The synergistic metabolic effects among the MetS components also increase the carcinogenesis risk in such patients [11]. For instance, Chen et al. associated alpha-fetoprotein levels with an increased risk of MetS [12]. Du et al. also associated serum carbohydrate antigen 19-9 with an increased risk of prevalent T2DM and MetS [13]. Furthermore, Kim et al. demonstrated a positive association between MetS and elevation of serum carcinoembryonic antigen [14]. These previous studies all confirm the potential carcinogenesis in individuals with MetS.

Human epididymis protein 4 (HE4) is a member of the whey acidic four-disulfide core family, and the WFDC2 gene encodes it in humans [15]. The HE4 gene is expressed in several normal tissues, including the human epididymis, trachea, salivary gland, lung, prostate, and kidney [16]. Numerous studies have shown that HE4 was a valuable tumor marker for the early diagnosis of ovarian malignancies [15,17,18]. Moreover, Lee et al. indicated that HE4 overexpression induces chemo resistance. Therefore, HE4 overexpression is associated with a worse prognosis for epithelial ovarian carcinoma (EOC) [19]. Furthermore, HE4 is included in the risk of ovarian malignancy algorithm (ROMA) value, indicating a high risk of finding EOC with values of 1.14 or greater [20]. In summary, HE4 is not only a tumor marker in the ROMA value but is also associated with chemo resistance and a worse prognosis in EOC.

Some studies associated each MetS component with HE4 levels, but none analyzed the relationship between MetS with HE4 levels [21]. Therefore, our study aimed to clarify the relationship of HE4 levels with MetS in the US general population according to the NHANES database.

## 2. Materials and Methods

### 2.1. Study Population

We selected adults (age ≥ 20 years) from the NHANES 2001–2002 who underwent detailed household interviews, including demographic and socioeconomic information, nutritional assessments, and physical examinations carried out by trained medical staff. The NHANES is a multi-stage, nationally representative, cross-sectional study carried out and approved by the US NCHS [22]. Continuous NHANES refers to the survey after 1999, when the survey moved to continuous data collection in two-year cycles. The laboratory data designed in NHANES 2001–2002 included males and females for serum triglycerides (TG), high-density lipoprotein (HDL), C-reactive protein, glycohemoglobin, etc. However, the laboratory data designed in NHANES 2001–2002 about serum HE4 and cancer antigen 125 (CA-125) were only for women [23]. The total number of participants was 11,039 (female number was 5887; serum HE4 collected only from female participants), and we enrolled 2104 female subjects for the final analyses. Our enrollments were only females aged ≥ 20 years because of the NHANES 2001–2002 study design regarding HE4 levels [23]. We excluded subjects with missing information, particularly about experiencing angina, coronary artery disease (CAD) history, smoking history, body mass index (BMI), MetS components, and values of serum creatinine, aspartate aminotransferase (AST), and HE4.

### 2.2. Definitions of Metabolic Syndrome

The definition of MetS in our study was based on the US NCEP Adult Treatment Panel III (ATP-III), which defined MetS if at least three out of five criteria are met: central adiposity/waist circumference ≥ 88 cm for women, systolic blood pressure (SBP) ≥ 130 mmHg or diastolic blood pressure (DBP) ≥ 85 mmHg, serum HDL ≤ 50 mg/dL for women, fasting blood sugar ≥ 100 mg/dL, and fasting TG ≥ 150 mg/dL [24].

### 2.3. Measurement: Covariates and Serum Human Epididymis Protein 4

Our study participants were interviewed to collect information, including age, race/ethnicity, smoking history, CAD history, BMI, AST (U/L), and serum creatinine (mg/dL). Blood sample analyses for HE4 were performed in the Genital Tract Biology Laboratory (Brigham and Women’s Hospital, Lab Director Fichorova) using the Meso Scale Discovery electrochemiluminescence immunoassay platform. The eligible samples were taken from female participants with consent forms for further studies from the NHANES study during 2001–2002. Quality control (QC) pools were prepared and split into multiple aliquots, and acceptable QC pool variation was set at 25%. The linearity range for HE4 was 3600–0.55 pM, and all samples were tested undiluted.

### 2.4. Statistical Analysis

Statistical analysis was done using the Statistical Product and Service Solutions, version 18.0 (SPSS Inc., Chicago, IL, USA). Continuous variables were presented as mean ± standard deviation, while nominal and ordinal data were presented as percentages. Demographic characteristics were compared using the ANOVA tests for normally distributed continuous variables, Kruskal–Wallis test for continuous variables without normally distributed, and the Chi-square test for discrete variables. Since HE4 levels were not normally distributed, a logarithmic transformation was performed to normalize the distributions of HE4 levels. The association of HE4 levels with presence of MetS and its components were assessed via multivariate analyses. Model 1 was not adjusted for other variables, whereas model 2 was adjusted for age, race/ethnicity, BMI, serum AST, creatinine, and history of CAD.

### 2.5. Ethics Statement

We derived the information in our study from the NHANES 2001–2002 database. The National Center for Health Statistics (NCHS) approved the study protocol; the approved date is 29 November 2017. The NCHS is part of the Centers for Disease Control and Prevention (CDC) and is responsible for producing national health statistics. Participants provided informed consent before undergoing data collection procedures and a series of health examinations.

## 3. Results

After the inclusion and exclusion criteria, this study analyzed 2104 female participants from the NHANES database. Table 1 shows the clinical characteristics of the study population. A total of 593 subjects met the MetS criteria, while there were 1511 individuals without MetS. In addition, there were 641 (30.5%), 1349 (64.1%), 721 (34.3%), 651 (30.9%), and 363 (17.3%) patients that met each ATP-III defined MetS criteria: high blood pressure, abdominal obesity, low HDL cholesterol, high triglycerides, and hyperglycemia, respectively. As shown in Table 1, HE4 levels were significantly higher among MetS subjects with HE4 levels of 25.05 ± 22.49 pM than those without MetS (*p* < 0.001). MetS participants also had significantly higher levels of fasting glucose, TG, AST, and creatinine, whereas those without MetS had lower SBP, DBP, waist, BMI, and age. Compared to the MetS group, significantly fewer individuals without MetS were diagnosed with CAD or chronic kidney disease (CKD).

Table 2 shows the regression coefficients of the presence and number of metabolic syndrome components for HE4 level. Table 2 shows the regression coefficients of the presence and number of metabolic syndrome components for HE4 level. There were influences of individual parameters on HE4 levels in model 1, such as age, race/ethnicity, BMI, serum AST and creatinine, history of CAD, and cigarette smoking. We adjusted for these covariates in model 2. The β coefficients of the HE4 levels of MetS individuals were 0.281 (95% confidence interval [CI]: 0.208–0.355, *p* < 0.001) in model 1 and 0.097 (95% CIs, 0.028–0.166, *p* = 0.006) in model 2. The β coefficients of HE4 levels of participants with 1, 2, 3, and ≥4 features of MetS in model 2 were 0.072 (95% confidence interval (CI): −0.015–0.159), 0.125 (95% CI: 0.030–0.220), 0.161 (95% CI: 0.053–0.270) and 0.242 (95% CI: 0.117–0.368), respectively, and all *p* values were all <0.001. Furthermore, the *p*-value for the trend was <0.001. These results demonstrated a significant association between MetS and HE4 levels in unadjusted and adjusted models. Furthermore, HE4 levels were positive associated with participants with 1, 2, 3, and ≥4 features of MetS among our study participants.

Table 3 shows that low HDL levels and high TG levels were significantly associated with HE4 levels in the adjusted models (*p* < 0.01). High blood pressure and high serum glucose levels were associated with HE4 levels before adjusting for covariates, but this became insignificant after adjusting for covariates. The last component of MetS, abdominal obesity, was not significantly associated with HE4 levels in both unadjusted and adjusted models.

## 4. Discussion

Among US female adults from the NHANES dataset, we found that the presence of MetS was significantly associated with HE4 levels. We also noticed a positive relationship between HE4 levels and an increased number of MetS components. These associations remained significant after adjusting for covariates. Particularly, low HDL levels and high TG levels had the strongest associations with HE4 levels among the other MetS components.

In a study by Zhang, serum HE4 was negatively correlated with HDL, positively correlated with blood pressure, but not correlated with TG and serum glucose in T2DM patients [21]. Huang et al. demonstrated that diabetes and dialysis patients had higher HE4 levels than healthy individuals. They also reported that high HE4 levels were associated with high SBP, but had no difference in TG, HDL, and fasting glucose levels [25]. Qu et al. identified age, postmenopausal status, and smoking habits as factors that potentially contribute to HE4 levels based on many other studies [26,27,28,29,30]. Furthermore, Bolstad et al. reported that, compared to the BMI of 20 individuals, 10% lower HE4 levels were at BMI of 30 individuals [26]. Collectively, the association among each MetS component with HE4 levels is inconclusive. However, HE4 levels were positively associated with age, postmenopausal status, and smoking habits but negatively associated with BMI. Thus, the covariates in our study included age, BMI, and smoking history.

The most prominent finding in our analysis was that the presence of MetS was significantly associated with HE4 levels (adjusted β coefficient: 0.097, 95% CI: 0.028–0.166, *p* = 0.006); there was also a positive relationship between HE4 levels and an increased number of MetS components. The adjusted β coefficients of HE4 levels of participants with 1, 2, 3, and ≥4 components of MetS were 0.072, 0.125, 0.161, and 0.242, respectively, and all *p* values were <0.001.

MetS is a condition with increased macrophage infiltration, which implies the involvement of interleukin 6 (IL-6) and tumor necrosis factor-alpha (TNF-α) [31]. Feingold et al. reported that IL-6 and TNF-α stimulate lipolysis and increase the flow of free fatty acids to the liver, which help induce hepatic TG synthesis and hypertriglyceridemia [32]. In addition, Bene et al. also reported that TNF-α upregulated HE4 expression with elevated IL-6 [33]. These mechanisms could explain the positive associations among HE4 levels, hypertriglyceridemia, and MetS that we found in our study. Our study also indicated that low HDL levels were significantly associated with HE4 levels. Nardo et al. indicated that HDL attenuates the toll-like receptor (TLR)-mediated inflammatory response in macrophages, releasing IL-6 and TNF [34]. Macpherson et al. also reported that HDL suppressed TLR2-induced TNF release in immunodeficient patients [35]. In summary, low HDL levels contribute to a TLR2-mediated pro-inflammatory status, leading to high HE4 levels.

On the other hand, HE4 levels significantly positively correlated with blood pressure and hyperglycemia, but this relationship was not significant after adjusting for covariates. Numerous studies have shown that HE4 levels were positively associated with creatinine levels [26,36,37]. In our study, a higher percentage of individuals with MetS was diagnosed with CKD (*p* < 0.001) and had higher creatinine levels (*p* < 0.008). These findings may indicate that the elevated creatinine levels resulting from hypertension or hyperglycemia had a more significant influence on HE4 levels.

The serum HE4 level, a better diagnostic biomarker than CA-125 in EOC patients, were included in the ROMA score to differentiate benign ovarian tumors from EOC [26,38,39]. A previous study found that HE4 levels had similar sensitivity but higher specificity than CA125 for diagnosing recurrent EOC [40]. Our study showed a significant association between the presence of MetS and HE4 levels and a linear increase in HE4 levels associated with the number of MetS components. These results from NHANES database may suggest a positive relationship between MetS and EOC in female individuals. However, Bjørge et al. reported that there was no overall association between MetS and ovarian cancer risk. They also indicated that increased cholesterol levels, blood pressure, and BMI increased the risks of mucinous and endometrioid tumors and EOC mortality in women aged ≥50 years [41].

Moreover, Michels et al. indicated that women meeting the MetS criteria had a reduced EOC risk; and impaired fasting glucose was also associated with reduced risk (odds ratio: 0.90, 95% CI: 0.87–0.93) [42]. They also showed that compared to women without any MetS components, having 1–2 syndrome components was associated with increased EOC risk, but having ≥3 syndrome components was not. In summary, the relationship of MetS with EOC is inconclusive. However, our study might suggest a positive correlation between MetS and EOC, based on a strong linear increase in HE4 levels with an increase in MetS components.

However, this study still has several limitations. Firstly, we could only measure serum HE4 levels in a subset of patients from the NHANES dataset who had baseline plasma samples available. Our eligible samples were only from female participants because of the NHANES 2001–2002 study design regarding HE4 levels. Thus, we were unable to analyze the associations between HE4 levels and MetS in male participants. Based on the previous studies included both sexes as mentioned earlier, there were positive associations among HE4 levels, hypertriglyceridemia, and MetS whereas, there was a negative association between HDL and HE4 levels [31,32,33,34,35]. Thus, it is possible to infer a significant association between the presence of MetS and HE4 levels in males. In addition, it is also possible to infer low HDL levels and high TG levels are associated with HE4 levels in males. Secondly, there may have been some aspects of HE4 levels not included in our covariates. For example, Kluz et al. analyzed HE4 concentration during the various stages of pregnancy and found that HE4 is lowest during the first trimester pregnancies [43]. Lastly, this is a retrospective cross-sectional study, and further prospective studies include both sexes are required to confirm our findings.

## 5. Conclusions

In conclusion, our findings suggested a significant association between the presence of MetS and HE4 levels. HE4 levels were positively related to an increased number of MetS components (with 1, 2, 3, and ≥4 features of MetS, all *p* < 0.001). Among the MetS components, low HDL levels and high TG levels were independently associated with HE4 levels.

## Figures and Tables

**Table 1:** Characteristics of participants with or without metabolic syndrome.

Variables	Metabolic Syndrome		*p* Value
	Yes (*n* = 593)	No (*n* = 1511)	
^a^ Continuous variables, mean ± SD			
HE4 (pM)	25.05 ± 22.49	18.86 ± 22.87	<0.001
Age (year)	56.83 ± 17.66	43.90 ± 18.22	<0.001
Fasting glucose (mg/dL)	110.66 ± 44.33	86.13 ± 16.49	<0.001
Waist (cm)	104.47 ± 13.51	90.50 ± 14.27	<0.001
Triglyceride (mg/dL)	211.11 ± 120.65	107.94 ± 58.52	<0.001
HDL-C (mg/dL)	47.55 ± 13.34	61.81 ± 15.57	<0.001
Systolic blood pressure (mmHg)	139.16 ± 24.23	120.44 ± 21.49	<0.001
Diastolic blood pressure (mmHg)	72.21 ± 15.38	68.80 ± 12.35	<0.001
BMI (kg/m^2^)	31.78 ± 6.52	26.96 ± 6.17	<0.001
AST (U/L)	23.08 ± 10.89	21.71 ± 8.00	0.001
Creatinine (mg/dL)	0.82 ± 0.31	0.76 ± 0.48	0.008
^b^ Categorical variables (%)			
Race			
Non-Hispanic white	52.4	54.9	0.052
Non-Hispanic black	15.3	17.8	0.052
Other	3.7	3.6	0.052
Smoking	42.0	38.9	0.326
Ever had diagnosis			
Coronary heart disease	4.4	2.2	0.002
Chronic kidney disease	6.2	2.1	<0.001

^a^ Continuous variables were compared using Student’s *t*-test. ^b^ Categorical variables were compared using Chi-square or Fisher’s exact test and expressed as no. (%). Definition of abbreviations: HE4 = human epididymal protein 4, HDL-C = high-density lipoprotein cholesterol, BMI = body mass index, AST = Aspartate transaminase.

**Table 2:** Regression coefficients of the presence and number of metabolic syndrome components for human epididymal protein 4 level.

Variables	Model 1		Model 2	*p* Value
	β (95% CI)	*p* Value	β (95% CI)	
Presence of metabolic syndrome	0.281 (0.208, 0.355)	<0.001	0.097 (0.028, 0.166)	0.006
Number of metabolic syndrome components				
1	0.121 (0.023, 0.219)	0.016	0.072 (−0.015, 0.159)	<0.001
2	0.149 (0.050, 0.248)	0.003	0.125 (0.030, 0.220)	<0.001
3	0.320 (0.211, 0.429)	<0.001	0.161 (0.053, 0.270)	<0.001
4 or 5	0.484 (0.357, 0.610)	<0.001	0.242 (0.117, 0.368)	<0.001
*p* for trend	<0.001		<0.001	

Model 1 = unadjusted. Model 2 = Model 1 + age, race-ethnicity, BMI, serum AST and creatinine, history of coronary heart disease, cigarette smoking. Definition of abbreviations: BMI = body mass index, AST = Aspartate transaminase.

**Table 3:** Regression coefficients of each metabolic syndrome component for human epididymal protein 4 level.

Variables	Model 1		Model 2	*p* Value
	β (95% CI)	*p* Value	β (95% CI)	
Components of metabolic syndrome				
Abdominal obesity	−0.056 (−0.168, 0.057)	0.332	−0.077 (−0.171, 0.016)	0.105
High blood pressure	0.439 (0.369, 0.509)	<0.001	0.037 (−0.036, 0.110)	0.317
High triglycerides	0.172 (0.100, 0.243)	<0.001	0.107 (0.045, 0.168)	0.001
Low HDL-C	0.060 (−0.010, 0.130)	0.095	0.105 (0.045, 0.165)	0.001
Hyperglycemia	0.274 (0.186, 0.363)	<0.001	0.059 (−0.021, 0.139)	0.146
*p* for trend	<0.001		<0.001	

Model 1 = unadjusted. Model 2 = Model 1 + age, race-ethnicity, BMI, serum AST and creatinine, history of coronary heart disease, cigarette smoking. Definition of abbreviations: HDL-C = high-density lipoprotein cholesterol, BMI = body mass index, AST = Aspartate transaminase.

## Data Availability

The data that support the findings of this study are available from the corresponding author upon reasonable request.

## References

[1] Bovolini A., Garcia J., Andrade M.A., Duarte J.A. (2021). Metabolic Syndrome Pathophysiology and Predisposing Factors. Int. J. Sports Med..

[2] Rosa S., Arcidiacono B., Chiefari E., Brunetti A., Indolfi C., Foti D.P. (2018). Type 2 Diabetes Mellitus and Cardiovascular Disease: Genetic and Epigenetic Links. Front. Endocrinol..

[3] Fahed G., Aoun L., Zerdan M.B., Allam S., Zerdan M.B., Bouferraa Y., Assi H.I. (2022). Metabolic Syndrome: Updates on Pathophysiology and Management in 2021. Int. J. Mol. Sci..

[4] Welty F.K., Alfaddagh A., Elajami T.K. (2016). Targeting inflammation in metabolic syndrome. Transl. Res..

[5] Hirode G., Wong R.J. (2020). Trends in the Prevalence of Metabolic Syndrome in the United States, 2011–2016. JAMA.

[6] Seyfried T.N., Flores R.E., Poff A.M., D’Agostino D.P. (2014). Cancer as a metabolic disease: Implications for novel therapeutics. Carcinogenesis.

[7] Esposito K., Chiodini P., Colao A., Lenzi A., Giugliano D. (2012). Metabolic syndrome and risk of cancer: A systematic review and meta-analysis. Diabetes Care.

[8] Puente D., López-Jiménez T., Cos-Claramunt X., Ortega Y., Duarte-Salles T. (2019). Metabolic syndrome and risk of cancer: A study protocol of case-control study using data from the Information System for the Development of Research in Primary Care (SIDIAP) in Catalonia. BMJ Open.

[9] Reuter S., Gupta S.C., Chaturvedi M.M., Aggarwal B.B. (2010). Oxidative stress, inflammation, and cancer: How are they linked?. Free Radic. Biol. Med..

[10] Carrier A. (2017). Metabolic Syndrome and Oxidative Stress: A Complex Relationship. Antioxid. Redox Signal..

[11] Cowey S., Hardy R.W. (2006). The metabolic syndrome: A high-risk state for cancer?. Am. J. Pathol..

[12] Chen Y., Zhao Y., Feng L., Zhang J., Zhang J., Feng G. (2016). Association between alpha-fetoprotein and metabolic syndrome in a Chinese asymptomatic population: A cross-sectional study. Lipids Health Dis..

[13] Du R., Cheng D., Lin L., Sun J., Peng K., Xu Y., Xu M., Chen Y., Bi Y., Wang W. (2017). Association between serum CA 19-9 and metabolic syndrome: A cross-sectional study. J. Diabetes.

[14] Kim K.N., Joo N.S., Je S.Y., Kim K.M., Kim B.T., Park S.B., Cho D.Y., Park R.W., Lee D.J. (2011). Carcinoembryonic antigen level can be overestimated in metabolic syndrome. J. Korean Med. Sci..

[15] Speeckaert M.M., Speeckaert R., Delanghe J.R. (2013). Human epididymis protein 4 in cancer diagnostics: A promising and reliable tumor marker. Adv. Clin. Chem..

[16] Galgano M.T., Hampton G.M., Frierson H.F. (2006). Comprehensive analysis of HE4 expression in normal and malignant human tissues. Mod. Pathol..

[17] Jia L.T., Zhang Y.C., Li J., Tian Y., Li J.F. (2016). The role of human epididymis protein 4 in the diagnosis of epithelial ovarian cancer. Clin. Transl. Oncol..

[18] Chang X., Ye X., Dong L., Cheng H., Cheng Y., Zhu L., Liao Q., Zhao Y., Tian L., Fu T. (2011). Human epididymis protein 4 (HE4) as a serum tumor biomarker in patients with ovarian carcinoma. Int. J. Gynecol. Cancer.

[19] Lee S., Choi S., Lee Y., Chung D., Hong S., Park N. (2017). Role of human epididymis protein 4 in chemoresistance and prognosis of epithelial ovarian cancer. J. Obstet. Gynaecol. Res..

[20] Moore R.G., Jabre-Raughley M., Brown A.K., Robison K.M., Miller M.C., Allard W.J., Kurman R.J., Bast R.C., Skates S.J. (2010). Comparison of a novel multiple marker assay vs the Risk of Malignancy Index for the prediction of epithelial ovarian cancer in patients with a pelvic mass. Am. J. Obstet. Gynecol..

[21] Zhang M., Zhao B., Xie J., Liang Y., Yang Z. (2019). Serum Human Epididymis Protein 4 is Associated with Renal Function and Diabetic Kidney Disease in Patients with Type 2 Diabetes Mellitus. BioMed Res. Int..

[22] Zipf G., Chiappa M., Porter K.S., Ostchega Y., Lewis B.G., Dostal J. (2013). National health and nutrition examination survey: Plan and operations, 1999–2010. Vital Health Stat..

[23] Cramer D.W., Vitonis A.F., Sasamoto N., Yamamoto H., Fichorova R.N. (2021). Epidemiologic and biologic correlates of serum HE4 and CA125 in women from the National Health and Nutritional Survey (NHANES). Gynecol. Oncol..

[24] (2001). Executive Summary of The Third Report of The National Cholesterol Education Program (NCEP) Expert Panel on Detection, Evaluation, And Treatment of High Blood Cholesterol In Adults (Adult Treatment Panel III). JAMA.

[25] Huang Y., Jiang H., Zhu L. (2020). Human Epididymis Protein 4 as an Indicator of Acute Heart Failure in Patients with Chronic Kidney Disease. Lab. Med..

[26] Bolstad N., Øijordsbakken M., Nustad K., Bjerner J. (2012). Human epididymis protein 4 reference limits and natural variation in a Nordic reference population. Tumour. Biol..

[27] Qu W., Li J., Duan P., Tang Z., Guo F., Chen H., Zhu X., Jiang S.W. (2016). Physiopathological factors affecting the diagnostic value of serum HE4-test for gynecologic malignancies. Expert Rev. Mol. Diagn..

[28] Hertlein L., Stieber P., Kirschenhofer A., Krocker K., Nagel D., Lenhard M., Burges A. (2012). Human epididymis protein 4 (HE4) in benign and malignant diseases. Clin. Chem. Lab. Med..

[29] Moore R.G., Miller M.C., Eklund E.E., Lu K.H., Bast R.C., Lambert-Messerlian G. (2012). Serum levels of the ovarian cancer biomarker HE4 are decreased in pregnancy and increase with age. Am. J. Obstet. Gynecol..

[30] Tian Y., Wang C., Cheng L., Zhang A., Liu W., Guo L., Ye H., Huang Y., Chen J., Ou Q. (2015). Determination of reference intervals of serum levels of human epididymis protein 4 (HE4) in Chinese women. J. Ovarian Res..

[31] Mohammadi M., Gozashti M.H., Aghadavood M., Mehdizadeh M.R., Hayatbakhsh M.M. (2017). Clinical Significance of Serum IL-6 and TNF-α Levels in Patients with Metabolic Syndrome. Rep. Biochem. Mol. Biol..

[32] Feingold K.R., Doerrler W., Dinarello C.A., Fiers W., Grunfeld C. (1992). Stimulation of lipolysis in cultured fat cells by tumor necrosis factor, interleukin-1, and the interferons is blocked by inhibition of prostaglandin synthesis. Endocrinology.

[33] Bene Z., Fejes Z., Szanto T.G., Fenyvesi F., Váradi J., Clarke L.A., Panyi G., Macek M., Amaral M.D., Balogh I. (2021). Enhanced Expression of Human Epididymis Protein 4 (HE4) Reflecting Pro-Inflammatory Status Is Regulated by CFTR in Cystic Fibrosis Bronchial Epithelial Cells. Front. Pharmacol..

[34] De Nardo D., Labzin L.I., Kono H., Seki R., Schmidt S.V., Beyer M., Xu D., Zimmer S., Lahrmann C., Schildberg F.A. (2014). High-density lipoprotein mediates anti-inflammatory reprogramming of macrophages via the transcriptional regulator ATF3. Nat. Immunol..

[35] Macpherson M.E., Halvorsen B., Yndestad A., Ueland T., Mollnes T.E., Berge R.K., Rashidi A., Otterdal K., Gregersen I., Kong X.Y. (2019). Impaired HDL Function Amplifies Systemic Inflammation in Common Variable Immunodeficiency. Sci. Rep..

[36] Wang L., Sun Y., Cai X., Fu G. (2018). The diagnostic value of human epididymis protein 4 as a novel biomarker in patients with renal dysfunction. Int. Urol. Nephrol..

[37] Yuan T., Li Y. (2017). Human Epididymis Protein 4 as a Potential Biomarker of Chronic Kidney Disease in Female Patients with Normal Ovarian Function. Lab. Med..

[38] Yanaranop M., Anakrat V., Siricharoenthai S., Nakrangsee S., Thinkhamrop B. (2017). Is the Risk of Ovarian Malignancy Algorithm Better Than Other Tests for Predicting Ovarian Malignancy in Women with Pelvic Masses?. Gynecol. Obstet. Investig..

[39] Romagnolo C., Leon A.E., Fabricio A.S.C., Taborelli M., Polesel J., Del Pup L., Steffan A., Cervo S., Ravaggi A., Zanotti L. (2016). HE4, CA125 and risk of ovarian malignancy algorithm (ROMA) as diagnostic tools for ovarian cancer in patients with a pelvic mass: An Italian multicenter study. Gynecol. Oncol..

[40] Lakshmanan M., Kumar V., Chaturvedi A., Misra S., Gupta S., Akhtar N., Rajan S., Jain K., Garg S. (2019). Role of serum HE4 as a prognostic marker in carcinoma of the ovary. Indian J. Cancer.

[41] Bjørge T., Lukanova A., Tretli S., Manjer J., Ulmer H., Stocks T., Selmer R., Nagel G., Almquist M., Concin H. (2011). Metabolic risk factors and ovarian cancer in the Metabolic Syndrome and Cancer project. Int. J. Epidemiol..

[42] Michels K.A., McNeel T.S., Trabert B. (2019). Metabolic syndrome and risk of ovarian and fallopian tube cancer in the United States: An analysis of linked SEER-Medicare data. Gynecol. Oncol..

[43] Gasiorowska E., Kluz T., Lipski D., Warchoł W., Tykarski A., Nowak-Markwitz E. (2019). Human Epididymis Protein 4 (HE4) Reference Limits in Polish Population of Healthy Women, Pregnant Women, and Women with Benign Ovarian Tumors. Dis. Markers.

